# Bending the rules: curvature’s impact on cell biology

**DOI:** 10.1186/s12915-025-02416-3

**Published:** 2025-10-06

**Authors:** Carole Arnold, Ismail Tahmaz, Marie-Ly Chapon, Hasna Maayouf, Valeriy Luchnikov, Jean-Louis Milan, Fabricio Borghi, Laurent Pieuchot

**Affiliations:** 1 IS2M (CNRS - UMR 7361), Université de Haute-Alsace, 68100 Mulhouse, France; 2 Univ Gustave Eiffel, Aix Marseille Univ, LBA, 13016 Marseille, France; 3 Aix Marseille Univ, CNRS, Marseille, ISM France; 4 Instituto de Fisica, Universidade Federal Do Rio de Janeiro, Rio de Janeiro, 21941-611 Brazil

**Keywords:** Curvature, Curvotaxis, Cell migration, Substrate topography, Mechanosensing

## Abstract

Curvature is a ubiquitous feature in biology, shaping structures at every scale and playing diverse roles in processes ranging from membrane dynamics to tissue organization. In this review, we first introduce briefly the fundamental concepts and mathematical principles of curvature. The second section explores how membrane curvature is perceived by molecular sensors and integrated into cellular responses. The third section examines the effects of curvature on cellular processes and behaviors at the cell-scale, providing a detailed discussion of the underlying mechanisms. Finally, we offer insights into emerging perspectives and highlight the future challenges in unraveling the multifaceted roles of curvature in biology.

## The fundamental concepts and mathematical description of curvature

Initially considered as a mere geometric property, curvature is no longer viewed as a passive feature, but as an active and dynamic element driving fundamental biological processes. Examples of curvature in the living world span from the molecular level (e.g., undulated collagen fibers, crescent-shaped BAR proteins, the thylakoid membrane system in chloroplasts) to organelles (e.g., cisternae of the endoplasmic reticulum, spherical vesicles with varying diameter, the membrane of chloroplasts) and cells (e.g., cylindrical axons of neurons, the cell wall of xylem vessels, helical/spiral/S-shape bacteria) [1]. The plasma membrane is of particular interest for this review, because its dynamic curvature—occurring both at local and larger cellular scales—directly shapes a range of vital biological processes. Continuous and regulated membrane bending is fundamental for functions such as vesicle trafficking, compartmentalization, and signal transduction, with changes in curvature actively influencing membrane organization, protein activity, and cellular behavior. Recently, the phenomenon of “curvotaxis” was described, in which cells migrate in response to substrate curvature [2].

**Fig. 1:**
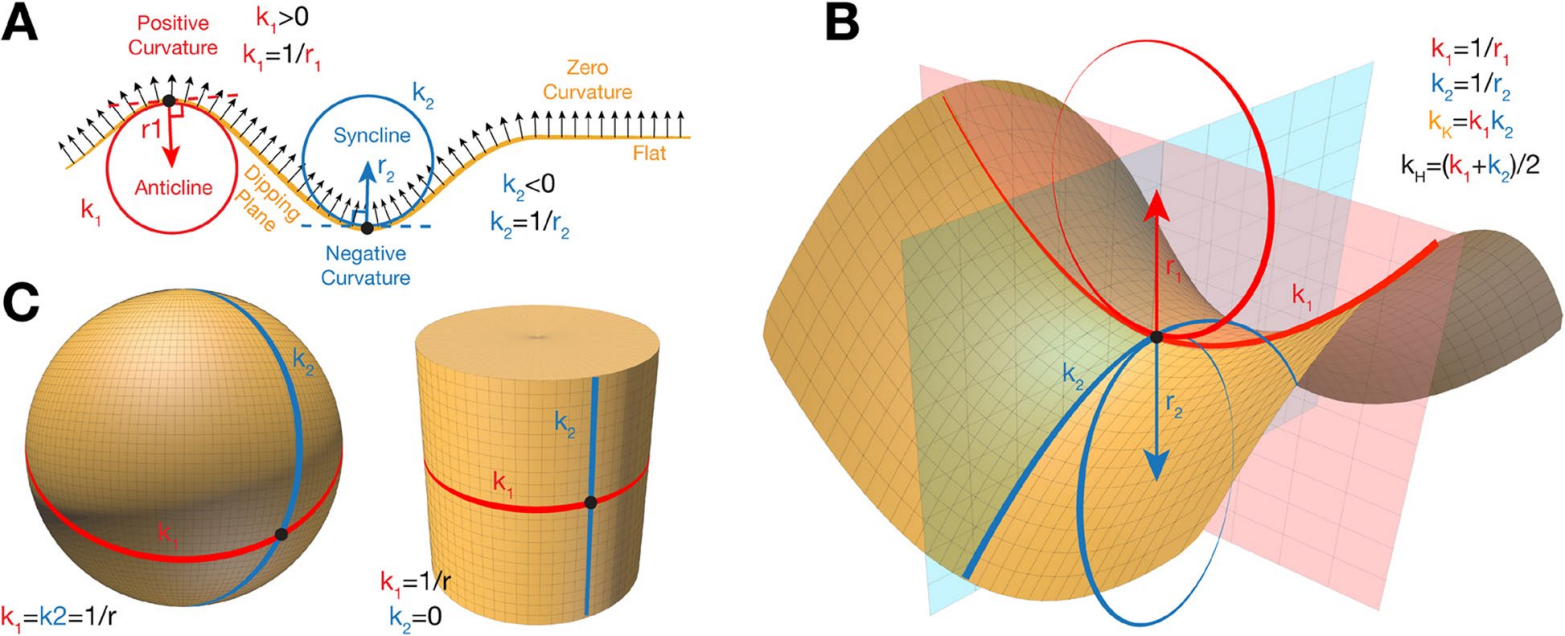
Fundamental concepts of surface curvature. **A** Convex region (positive curvature, in red), concave regions (negative curvature, in blue), flat regions (zero curvature); **B** a saddle-shaped region (negative Gaussian curvature K, with opposite principal curvatures), **C** a sphere (positive Gaussian and mean curvature with equal principal curvatures), and cylinder (zero Gaussian curvature with one principal curvature equal to zero, and positive mean curvature). K, Gaussian curvature; H, mean curvature; k1 and k2, principal curvatures

To mathematically describe curvature, researchers commonly use several parameters [1]. Local curvature refers to the bending at a specific point on a surface or curve, describing how much the shape deviates from being flat or straight at that location. For a 2D curve, it is given by κ = 1/R where R is the radius of the osculating circle at the point (Fig. 1A). For a surface, two principal curvatures (κ_1_ and κ_2_), which are respectively the maximum and minimum curvatures of the surface at that point, are measured along two mutually perpendicular directions (Fig. 1B). If both κ_1_ and κ_2_ are positive, the surface is locally convex. If both are negative, the surface is locally concave. A positive value of κ_1_ combined with negative κ_2_ defines a saddle-shaped surface. The mean curvature (H) describes the tendency of the surface to bend inwards or outwards at a point and is the average of the two principal curvatures, calculated as H = (κ_1_ + κ_2_)/2. The Gaussian curvature (K) at a point on a surface measures the bending in different directions and is defined as the product of the two principal curvatures: K = κ_1_·κ_2_. All these parameters are effectively used to describe both basic and complex 3D shapes encountered at all scales in biological systems, such as spheres, tubes, waves, saddles, and hyperboloids (Fig. 1C). In the following sections, we will show how curvature is crucial in shaping the structure and function of membranes, organelles, and cells.

## The molecular effects of cell membrane curvature

The structure of biological cells inherently requires curved plasma membranes to define their boundaries and compartmentalize their interiors. This is essential to numerous biological processes including cell motility, polarity, signaling, and division. Additionally, dysregulation of membrane curvature has been implicated in various diseases such as cancer, cardiovascular issues, and neurodegeneration [3, 4]. Studies have employed sophisticated tools to investigate the precise impacts of membrane curvature on cellular biology and physiology, including artificially curved isolated membranes on solid substrates, and giant unilamellar vesicles (GUVs)—vesicles engineered to become a single lipid bilayer in a spherical shape and that are physiologically similar to plasma membranes (Table 1).

**Table 1:** Model membranes for studying curvature-induced molecular effects

Researchers have generated models of lipid bilayers to gain insights into the biophysical principles governing membrane curvature and its role in cellular processes such as membrane trafficking, signaling, and morphogenesis. These models enable detailed investigations into curvature-sensitive phenomena under well-defined experimental conditions. The most commonly used systems are vesicles, either isolated from cells by fractionation or synthesized by mixing lipids in water, then subsequently engineered to obtain a single lipid bilayer (“unilamellar” membrane). These are relatively easy to fabricate (spontaneous assembly) and display a spherical shape with different degrees of curvature due to variable diameter. They come in three flavors, the small (SUVs, 50–200 nm), the large (LUVs, 200 nm–1 μm), and the giant unilamellar vesicles (GUVs, 1–100 µm). GUVs are physiologically similar to plasma membranes, while SUVs recapitulate intracellular vesicles. All variants can be labeled through incorporation of fluorescent molecules, although only LUVs and GUVs are amenable to imaging, the SUVs being too small. All can be combined with micropipette aspiration for membrane deformation and control over curvature Less commonly used, the supported lipid bilayers (SLBs) typically exhibit a planar morphology with a surface area ranging from several square micrometers to millimeters. A lipid solution containing cholesterol and phospholipids, such as phosphatidylcholine or phosphatidylserine, is mixed in an organic solvent, such as chloroform or ethanol, and spread on a solid substrate (e.g., PDMS made hydrophilic by O_2_ plasma treatment). The lipids spontaneously self-assemble into bilayers upon solvent evaporation. SLBs have good stability and can also be fluorescently labeled. Due to planar shape, they bear less resemblance to natural cellular membranes compared to vesicles, but their great benefit over those is their compatibility to mechanical probing techniques (e.g., atomic force microscopy [AFM], quartz crystal microbalance [QCM]) and some advanced techniques requiring a rigid surface (e.g., total internal reflection fluorescence microscopy [TIRF], surface plasmon resonance [SPR]). Moreover, the rigid surface utilized for in SLB fabrication can easily be customized to incorporate different curvatures. Finally, other methodologies for investigating curvature include bilayer membrane tubes and lipid-coated nanoparticles or nanodiscs.	

### How membrane curvature impinges on the distribution and activity of proteins, lipids, and ions

**Fig. 2:**
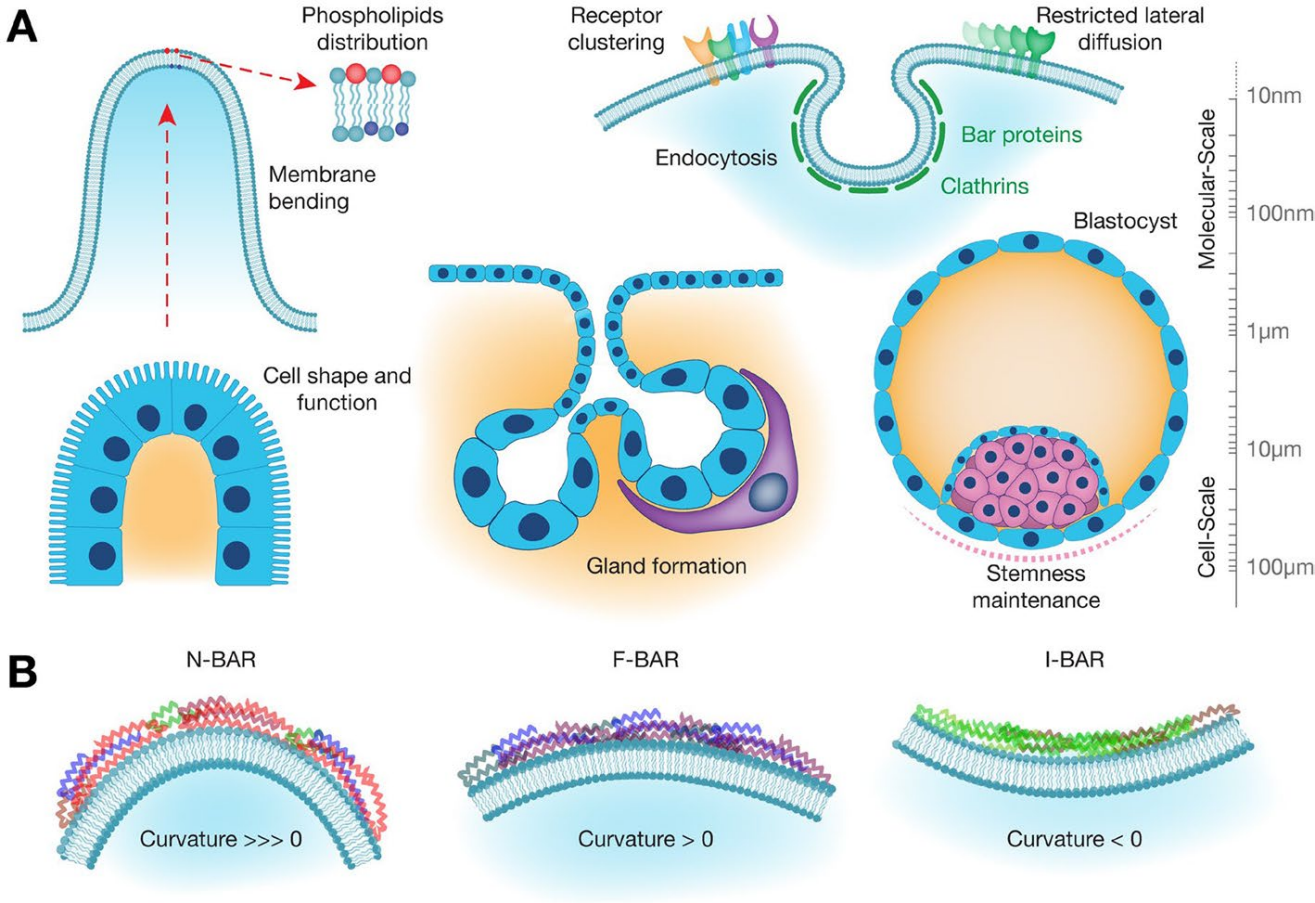
Multiscale regulation of membrane and cellular dynamics through curvature. **A** Molecular-scale curvature influences lipid/protein mobility, vesicular transport, and membrane remodeling via curvature-sensing proteins (e.g., BAR domains), lipid asymmetry, and mechanosensitive ion channels. Cell-scale curvature governs global cellular behavior, including shape/orientation, adhesion dynamics, migration (via behavior like curvotaxis), and fate decisions such as proliferation and differentiation. **B** The BAR-domain superfamily exhibits structural variation in length and curvature that promotes membrane-binding specificity: N-BAR and F-BAR domains preferentially target positively curved membranes, with N-BAR domains sensing greater positive curvature, whereas I-BAR domains adopt an inverted architecture that favors negatively curved membranes

Cell membrane curvature affects the spatial organization of surface receptors and signaling proteins, which can influence perception of extracellular cues and signal transduction in the cytoplasm. Curvature can promote the formation of concentration gradients in membrane-associated molecules. In regions of high curvature (high kappa values), molecules may accumulate or be excluded, leading to spatial variations in their concentrations along the membrane [5–7] (Fig. 2A, upper right, receptor clustering). This has been shown so far for a limited number of integral proteins, membrane lipids, and the calcium ion, as described below.

#### Clustering and conformational changes of integral membrane proteins

The curvature of the cell membrane can significantly influence the distribution and function of membrane proteins. Proteins that preferentially interact with curved membranes may aggregate in regions of high curvature, leading to the formation of signaling complexes or receptor clusters, e.g., the pleiotropic glycolipid receptors GM_1_ and GT_1_b for toxins, viruses, and signaling molecules [8] or the three prototypic class A G protein-coupled receptors (GPCRs) [5]. The precise spatial arrangement, or sorting, of GPCRs is critical for their biological activity and frequently occurs within specialized membrane structures characterized by high positive H values, such as filopodia, endocytic pits, trafficking vesicles, or endosome tubules. Membrane curvature promotes oligomerization and thus alters the G protein-coupled photoreceptor rhodopsin activity [9]. Unlike GPCRs, the mechanosensitive ion channel Piezo1 tends to avoid highly curved membrane protrusions, such as filopodia, and may instead preferentially localize to regions of negative curvature, such as membrane invaginations [10]. Membrane curvature can generate mechanical constraints that influence the conformational states of membrane-associated proteins. In particular, disordered proteins or proteins with unstructured domains often interact with membranes through amphipathic regions or lipid-binding motifs. When the membrane bends, these interactions may be altered due to changes in lipid packing, surface area, or local tension. This can, in turn, affect the structural flexibility and functional state of the protein. [11]. Changes in membrane curvature can alter the accessibility of binding sites or induce conformational changes in proteins, thereby modulating their activity and downstream signaling (see [4] for review).

#### Lipid distribution

Membrane curvature can also influence lipid sorting and microdomain formation [12] (Fig. 2A, upper left, phospholipid distribution). Curved membranes may preferentially segregate specific lipid species or lipid microcompartments, which can in turn recruit or exclude certain membrane-associated proteins, as demonstrated for the membrane-anchored Ras small GTPases [13]. Interestingly, Ras exhibits isoform-specific sensitivity to membrane curvature, with one isoform preferring low curvatures, while the other has an affinity for high curvature membranes [14]. This arises from distinct membrane-anchoring motifs. K-Ras, with its farnesyl group and polybasic hypervariable region, preferentially associates with flat membranes enriched in phosphatidylserine via electrostatic interactions. In contrast, H-Ras, which is farnesylated and doubly palmitoylated, targets more curved membrane domains, such as lipid rafts, where its hydrophobic anchors insert more efficiently. Additionally, isoform-specific orientations of the G-domain relative to the membrane further modulate curvature sensitivity and effector accessibility.

Curvature impacts the distribution of lipids over the two leaflets of the membrane bilayer [15]. Researchers have placed lipid membranes on shaped surfaces to show that curvature elasticity (i.e., the elastic resistance of a membrane to changes in its curvature) plays a role in the distribution of lipids over membrane leaflets [8]. As demonstrated by computational studies, membrane bending can be influenced not only by lipid composition but also by differences in lateral tension between the two leaflets, arising from asymmetry in the lipid bilayer [16].

#### Ions and membrane curvature

Besides the described effects on proteins and lipids, some downstream players may also be affected by curvature. This is the case for Ca^2+^ ions, which preferentially bind to the concave face (with negative H value) of lipid bilayers as demonstrated in a modeling study [17]. This is a result obtained from a simulation study, which has not been observed in living cells. A single-vesicle assay with GUVs demonstrated that Ca^2+^ ions generate positive spontaneous curvature in negatively charged membranes, likely due to electrostatic repulsion between the ions, which drives outward bending to reduce their proximity. In contrast, Na^+^ ions effectively suppress this curvature effect. This regulatory role of Na^+^ may help maintain membrane stability and prevent unwanted budding or tubulation during signal transduction when intracellular Ca^2+^ levels rise [18]. However, the sign of curvature is controversial, with another study reporting a negative spontaneous curvature in the same conditions [19]. Moreover, alkali chlorides and particularly LiCl have been shown to curve biomembranes negatively, reaching values similar to those induced by certain proteins [20].

Overall, cell membrane curvature plays a critical role in shaping the spatial organization of surface receptors and signaling proteins, ultimately influencing various cellular processes such as signal transduction, cell adhesion, and membrane trafficking.

### The impact of membrane curvature on endocytosis and exocytosis

Cell membrane curvature also plays a role in regulating endocytosis and exocytosis by facilitating the formation of membrane vesicles (Fig. 2A, upper right, endocytosis). Components of the endocytic and exocytic machinery display distinct sensitivities to membrane curvature, with some proteins preferentially recognizing or stabilizing curved membranes, while closely related counterparts show little or no such interaction. While extensive research has elucidated how proteins involved in clathrin-mediated endocytosis (CME), such as endophilin, epsin, BAR domain proteins, AP180, FCho2, actin, dynamin, CALM, and clathrin, actively shape plasma membrane curvature [21–31], investigations into the reverse process—how plasma membrane curvature influences the activities of endocytic proteins—are relatively limited. However, it has been proved that central proteins involved in CME, namely clathrin and dynamin, exhibit a pronounced affinity for positive membrane curvatures characterized by a radius of less than 200 nm, which was not the case for non-CME endocytic protein caveolin-1 [32]. Another seminal study also revealed that induced membrane curvature via U-shaped nanopatterns recruits endocytic machinery and restores cargo activity in the absence of clathrin or FCHo1/2, but not AP2 [31]. Moreover, clathrin has generally been considered to have a central structural role in driving membrane invagination and vesicle formation (scaffold assembly, membrane deformation, cargo selection, vesicle formation, and disassembly after budding), but this role is challenged by findings showing that, under conditions of sustained membrane curvature, clathrin is not primarily required as a scaffold for recruiting other CME proteins. Further studies will be needed to refine our understanding of how membrane curvature contributes to endo- and exocytosis, particularly beyond the well-characterized role of protein scaffolding in clathrin-mediated endocytosis (CME). In particular, the extent to which curvature-induced membrane bending directly facilitates vesicle budding remains to be fully elucidated. Advanced computational and theoretical modeling approaches offer deeper insights into the underlying biophysical mechanisms, helping to elucidate how curvature dynamics integrate with molecular interactions to orchestrate these essential cellular processes [33, 34].

### The mechanisms of membrane curvature sensing: towards the concept of “curved adhesions”

The influence of curvature on numerous cellular processes further raises the question of how cells detect and respond to variations in membrane curvatures. The Bin/amphiphysin/Rvs (BAR) domain proteins enable membrane curvature sensing. The family comprises several members with distinct BAR domains varying in length and intrinsic curvature, and these members display varying binding affinity for different membrane curvatures (Fig. 2B). Indeed, N-BAR and F-BAR proteins preferentially bind to positively curved membranes (convex surfaces). These proteins contain crescent-shaped domains that match and stabilize outward membrane curvatures, and they play crucial roles in membrane tubulation, endocytosis, and cytoskeletal interactions. In contrast, I-BAR proteins favor negatively curved membranes (concave surfaces). I-BAR domains are structurally inverted and preferentially associate with inwardly curved membranes, such as those involved in filopodia formation (for a comprehensive discussion, please see [35]). While BAR domain proteins are capable of sensing curvatures within the nanometer scale, the recognition of micron-scale curvature is mainly mediated by the septin cytoskeleton [36]. Septins are fibrillar proteins forming hetero-oligomeric complexes that polymerize into filaments, which can further assemble into higher-order structures such as rings, gauzes (i.e., net-like structures), and filaments. These structures contribute to the formation of scaffolds that help organize membranes and define subcellular compartments, but also assist cells in sensing their local geometry or shape. Recently, Zhang et al. have used surfaces coated with nanopillars to induce positive membrane mean curvature at the nanoscale [37]. They identified integrin αvβ5 in adhesions forming on these curved regions, naming them “curved adhesions” to emphasize their importance in curvature sensing. Such adhesions recruit talin-1, paxillin, and zyxin but not vinculin or pFAK. These specific assemblies recruit a curvature-sensing protein FCHo2 (an F-BAR domain-containing protein) through interaction between the juxtamembrane region of integrin β5 and the microtubule-binding domain µHD of FCHo2, and they connect to F-actin other than thick stress fibers. Likewise, while clathrin is primarily known for its role in membrane curvature generation during endocytosis, recent research has revealed its additional critical function in focal adhesion dynamics [38]. This study demonstrated that clathrin accumulates at focal adhesions and departs alongside α5β1 integrin during disassembly, thereby facilitating focal adhesion turnover—an essential process for efficient cell migration.

Despite increasing research efforts aimed at understanding cellular membrane curvature sensing, and the discovery of specific adhesions on curvatures, our comprehension remains incomplete regarding the detailed molecular mechanisms, regulation of curvature sensing, integration with other cellular processes, and spatiotemporal dynamics.

## The pleiotropic effects of curvature at the cell-scale

At the microscale, curvature in cell monolayers arises during processes such as buckling, wrapping, bumping, budding, tubing, and gland formation. These dynamic structural changes result in specific cellular outcomes, including modulation of proliferation, migration, and differentiation, as cells respond to the mechanical and geometric cues provided by the curved microenvironment. Here we provide an overview of the key points.

### Cell migration

**Fig. 3:**
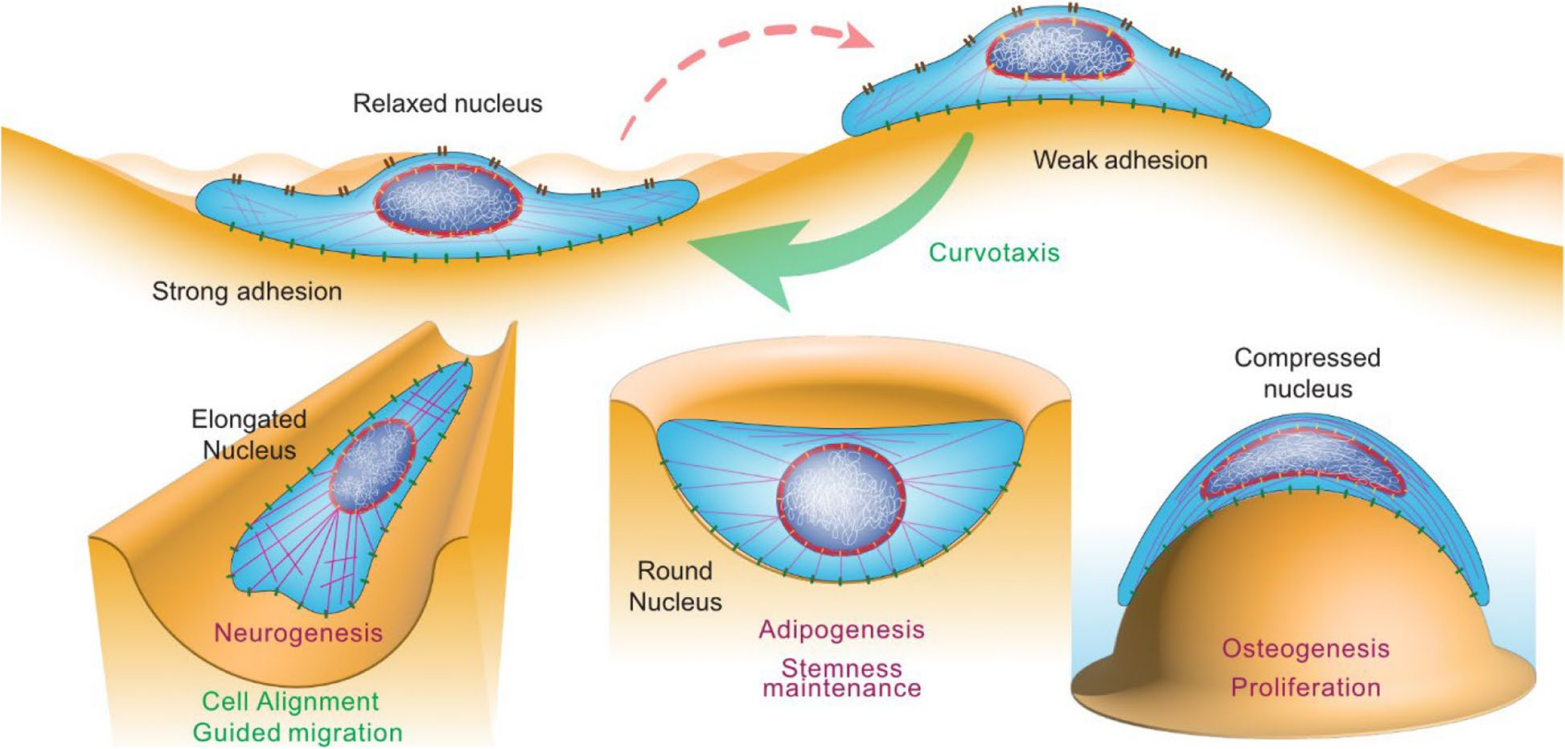
Cellular processes influenced by substrate curvature. This illustration depicts how substrate curvature dynamically modulates cellular morphology and function. Key effects include curvature-dependent alterations in cell and nuclear shape (e.g., elongation or compression), cytoskeletal reorganization (e.g., stress fiber alignment), and focal adhesion points distribution. Substrate curvature further guides migration patterns. These biophysical cues ultimately impact proliferation and cellular differentiation, including neurogenesis, adipogenesis, and osteogenesis

Cells can exhibit directional migration in response to curvature gradients in their environment, a process which was first identified in the social amoeba *Dictyostelium discoideum* and coined “curvotaxis” [39]. This ability to navigate curvatures has also been observed in mammalian cells [2]. Several cell types, such as fibroblasts, stem cells, and cancer cells [3], also exhibit migration preference towards concave regions (Fig. 3, upper part). All the underlying mechanisms of this intriguing phenomenon of curvotaxis have not yet been fully elucidated and the precise molecular details of curvature sensing mechanisms may vary among different cell types and contexts, but recent research has nonetheless identified several key processes, e.g., cytoskeletal organization, adhesion dynamics, and membrane tension. Cells migrating along concave surfaces often exhibit enhanced protrusion stability and directionality, possibly due to curvature-induced redistribution of integrins and actin regulators. However, not all cells respond similarly: for instance, fibroblasts and mesenchymal cells tend to align and migrate along concave or flat regions, while immune cells or amoeboid cells can go across convex or highly curved environments more easily, owing to their reduced dependence on adhesion and contractility. Thus, curvature may serve as a physical cue guiding cell migration in a cell-type- and context-dependent manner, influencing processes such as development, wound healing, or cancer invasion.

The potential to direct cell movement in a specific direction or over extended distances using a surface engineered with curvatures, thereby facilitating precise cell guidance, has been proposed. Manifacier et al. used computational modeling to explore this concept. They simulated the migration of individual cells on traveling wave patterns (defined as continuously alternating Gaussian values) with various dimensions and periodicity, elucidating the underlying mechanisms and cellular features involved. Their conclusions imply the potential to control cell migration with well-designed dynamic curved shapes [40].

### Influence of curvature on focal adhesion and actin organization and dynamics

Cells interact with extracellular matrix (ECM) components like collagen, fibronectin, and laminin via cell surface receptors called integrins. The binding of these proteins to ECM ligands triggers the formation of adhesion complexes that facilitate cell attachment and spreading on the substrate. Integrin clusters link actin filaments via large macromolecular assemblies named focal adhesions, containing adapter proteins (proteins like talin, vinculin, α-actinin, filamin, and tensin) and signaling proteins (focal adhesion kinase (FAK), Src, paxillin, and Rho GTPases) as well as structural proteins (like zyxin and VASP which help regulate the dynamics and organization of the actin cytoskeleton at those sites) [41]. Focal adhesions and associated structures are recognized as the primary structures that link the actin cytoskeleton to the ECM for transmission of mechanical forces. Cell migration can occur through the mechanism of actomyosin contraction, which is the contraction of the actin cytoskeleton through myosin molecular motors.

It has been recognized that substrate surface curvature affects cell shape as well as the spatial organization and dynamics of focal adhesions at the cell-substrate interface. Pieuchot et al. have systematically examined the distribution and size of focal adhesions in a series of wave patterns with various dimensions, emphasizing their importance in curvotaxis [2]. They used the FRET technique to demonstrate that focal adhesion abundance and persistence were dependent on the topography, with fewer and more stable adhesions in the concave areas, and conversely a greater number of integrin adhesions with lower lifetime in the convex regions. Bowers et al. cultured cells on poly(methyl methacrylate) (PMMA)-electrospun nanofibers displaying various controlled subcellular curvatures and showed that the arrangement of focal adhesions was affected with a predominant effect on vinculin and phosphorylation of FAK at tyrosine 925 [42]. This phosphorylation at Y925, mediated by Src family kinases, is known to reduce the affinity of the FAT domain for paxillin’s LD2 motif. This promotes paxillin dissociation and enables vinculin binding, contributing to FAK activation within focal adhesions in response to mechanical cues. Werner et al. showed that concave shapes (with negative principal curvatures) accelerated human mesenchymal stem cell (hMSC) migration compared to convex or flat ones, and that the cells modified their migration mode by forming elongated protrusions spanning over the pits. The attachment sites were located in the extensions, far from the cell’s center, and there was a reduction of the contact area between cell and surface, with cells in suspension above the wells [43]. By contrast, cells on convex or flat surfaces followed a more classical migration scheme with extension of the front edge, movement of the cell body, and withdrawal of the rear, with a full cell-surface contact. The forces at stake in all these migration processes have been reviewed elsewhere [44], using the molecular clutch model as a framework, in an analogy with a mechanical clutch in a car. In this model, the cytoskeleton of the cell acts as the “engine” that generates force, while the ECM serves as the “road” providing traction. The focal adhesions (FAs) between the cell and the ECM are akin to the clutch plates, engaging and disengaging to control the transmission of force. Another publication has proposed a biophysical model with complex chemomechanical feedback loops involving the generation of cellular forces, specific molecular flows such as actin or integrin, and the assembly of curved front motifs [45]. In this model, on concave surfaces, actin cable and actomyosin assembly are promoted, pulling cells forward. Conversely, on convex surfaces, focal adhesion formation is enhanced, enabling cell crawling.

Recent studies have highlighted how both membrane curvature and actin filament curvature influence actin organization and dynamics. For instance, membrane curvature has been shown to induce actin filament bundling [46] and bias the branching direction of Arp2/3-nucleated actin networks [47]. The actin network, in turn, generates forces that further deform the membrane, creating a self-reinforcing loop. In fact, the actin filament curvature itself biases branching direction, suggesting that cytoskeletal remodeling contributes to membrane shape changes [47]. Furthermore, curvature-driven cortical protein waves [48] and actin reorganization in response to nanoscale topography [49] underscore the intimate link between membrane shape and cytoskeletal remodeling. More recently, nanoscale condensation of N-WASP/FBP17 at curved membranes has been proposed as a mechanism for curvature-catalyzed actin nucleation [50]. These insights underscore the importance of mechanical forces and their regulation in cellular migration on substrates and provide a more integrated view of the biophysical and molecular processes at stake.

### Cell nucleus as curvature mechanosensor

There is a dynamic interplay between the cytoskeleton and the cell nucleus during curvotaxis (Fig. 3, upper part). Molecular connections between the cytoskeleton and the nuclear envelope facilitate communication and mechanical transmission between these two cellular compartments. Proteins such as linker of the nucleoskeleton and cytoskeleton (LINC) complexes span the nuclear envelope, connecting the nuclear lamina to the cytoskeleton. These complexes are formed by the interaction of SUN proteins on the nuclear envelope and nesprin proteins which connect to the cytoskeleton. These connections enable the transmission of forces from the cytoskeleton to the nucleus and vice versa, allowing the cell to sense and respond to mechanical signals [51].

Pieuchot et al. have shown that the LINC complex is necessary for curvotactic migration, playing a crucial role in transmitting mechanical cues from the cytoskeleton to the nucleus [2]. LINC notably transmits the forces from the cytoskeleton to the nucleus, orchestrating the dynamic interplay between the nucleus and the cytoskeleton, leading to nuclear deformation and finally to the migratory response. The nucleus might thus be considered as a topographical sensor and mechanical guide in the process of cell migration, in particular in the context of curvotaxis [52].

### Cellular contractile cortex, curved substrate, and nuclear positioning

Cell substrate curvature can affect nucleus shape and stiffness. As described above, Werner et al. showed that human mesenchymal stem cells (hMSCs) on convex curved substrates exhibited compressed, flattened nuclei with increased lamin-A expression, a key factor in nuclear stiffness. Conversely, concave substrates resulted in rounder nuclei and reduced lamin-A. They proposed that substrate curvature alters nuclear mechanical stress: convex surfaces increase basal compression due to a thinner cell profile and contractile actin cortex, whereas concave surfaces relieve stress by increasing cell thickness. This was computationally validated by Vassaux and Milan [53] using a model with a deformable nucleus, cytoplasm, and prestressed actin cortex that matched experimental cell stiffness data. The model replicated “curvotaxis” on sinusoidal substrates, with nuclei migrating into concave regions. Simulations revealed heterogeneous nuclear stress, with more compression over convex regions and relaxation over concave regions, biasing nuclear positioning. Migration simulations confirmed that cells prefer concave paths because overcoming convex compression requires more energy. Nuclear stiffness and actin contractility are critical for curvotaxis. Reducing lamin-A or disrupting actin abolished the curvature response. Computational studies [54] further confirmed that reduced nuclear stiffness or actin contractility prevented effective curvotaxis, mirroring the results of experimental lamin-A/F-actin inhibition. These results highlight nuclear mechanics as a key mediator of cellular responses to environmental curvature. This demonstrates the power of computational biophysics in understanding cell behavior on curved surfaces and highlights the role of nuclear mechanics in responding to mechanical cues.

### Gene expression changes and epigenetic regulation

**Fig. 4:**
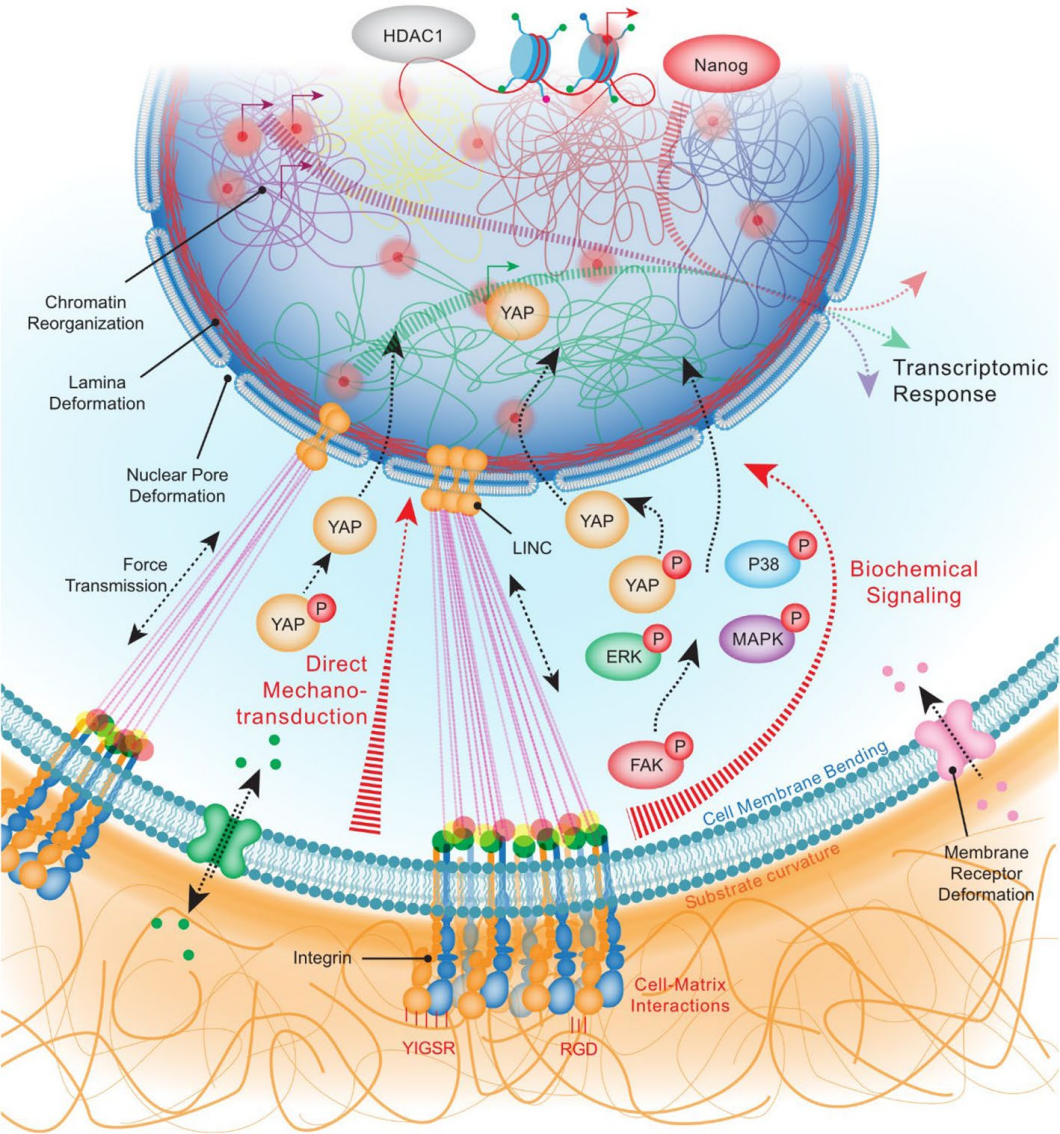
Coupling of ECM curvature to chromatin dynamics and transcriptional output through mechanotransduction pathways. ECM curvature sensed by integrin receptors may trigger genetic reprogramming through cytoplasmic signaling cascades, including ERK and MAPK pathways, as well as YAP dephosphorylation and nuclear translocation, driving transcriptional activation. Concomitantly, curvature-dependent forces transmitted via the LINC complex could alter nuclear lamina tension and chromatin architecture, influencing epigenetic states (e.g., histone modifications, chromatin accessibility) and downstream gene expression

It has been emphasized that nuclear-cytoskeletal interactions can influence gene expression patterns and cell fate decisions on curved substrates (Fig. 4). High-throughput approaches have already revealed some signaling pathways and genes [2]. The majority of the differentially regulated genes were those involved in cell differentiation, with others in cytoskeleton remodeling and cell proliferation. It remains uncertain whether mechanical forces applied to the nucleus on curved surfaces might alter chromatin conformation and accessibility, modulating the expression of genes involved in many processes, e.g., cell migration, adhesion, and polarity. However, studies have shown that convex curvature compresses the nucleus, leading to increased expression of lamin-A, a key component of the nuclear lamina. Lamin-A contributes to mechanical protection of DNA, enhances nuclear stiffness, and supports the ability of cells to sense and respond to curvature through curvotaxis [2, 43]. Moreover, lamin-A, through its interaction with lamina-associated domains, regulates various aspects of epigenetics like chromatin organization, gene expression regulation, DNA methylation, and epigenetic memory. Further investigation of chromatin dynamics in cells cultured on bended substratum might open a new avenue for the elucidation of gene expression and epigenetic changes in response to curvature.

### Minimization of energy

In recent years, an approach has been made to generate a theoretical “minimal cell” model to simplify the system and isolate physical factors (e.g., forces, energy) from their biochemical counterparts. This model features only a few basic elements: a closed vesicle containing curved membrane proteins linked to active cytoskeletal forces that self-organize to enable movement across a surface. This approach is intriguing for its potential to pinpoint the most significant factors in a given process and has led to the idea that minimizing the energy associated with membrane-substrate adhesion and the binding energy between membrane protein complexes might be critical factors influencing curvotaxis during cell migration [55]. Minimizing energy expenditure might help the cells to efficiently navigate curved paths, conserve metabolic resources, and optimize their response to external stimuli. This energy-efficient behavior could enhance cell survival and function, particularly in challenging microenvironments.

### Mechanotransduction

Mechanotransduction is the process by which cells convert a mechanical stimulus into biochemical activity and subsequently into cellular responses, such as changes in gene expression, cell behavior, and tissue organization. Like other physical cues (e.g., stiffness, mechanical forces), curvature can activate mechanotransduction processes in various cellular contexts [10, 56]. These involve both mechanical and biochemical mechanisms which interact and complement each other for mechanosensing (Fig. 4).

Direct mechanotransduction consists of the force transmission through the cytoskeleton directly to the nucleus and other organelles, without any involvement of intermediate biochemical signaling. Mechanical forces applied at focal adhesions can directly influence the tension and organization of the cytoskeleton, the cytoskeleton-nucleus connection through the LINC complex, and deform the nuclear envelope, causing changes in the positioning of chromosomes and nuclear components, chromatin organization, and modulation of gene expression. In the indirect mechanotransduction pathway, forces are initially sensed by cell surface receptors (such as integrins) and mechanosensitive ion channels (like Piezo channels or ion channels located on primary cilia) [10]. The stimulus is then converted into a biochemical cue through signaling cascades involving diverse kinases (e.g., FAK, ERK, PKA, AMPK, and CAMKII) and calcium ion flux (Fig. 4). One of the key players in the biochemical pathway is the Hippo/YAP signaling pathway. The core components of the Hippo pathway negatively regulate the activity of two effectors, the transcriptional co-activators Yes-associated protein (YAP) and transcriptional co-activator with PDZ-binding motif (TAZ) by stimulating their phosphorylation. Upon activation by various upstream signals including mechanical cues, unphosphorylated, active YAP/TAZ can translocate to the nucleus and bind to transcriptional enhanced associate domain (TEAD) transcription factors to regulate the expression of target genes involved in cell proliferation, survival, and differentiation [57].

Curvature sensing is a form of mechanosensing that activates the same pathways described above, involving mechanosensitive ion channels and membrane-associated proteins that sense changes in plasma membrane tension and transduce mechanical signals into the biochemical responses [10] (Fig. 4). Several studies have shown that curvature leads to YAP nuclear localization [2, 55, 58, 59]. The mechanism proposed to explain this observation is based on induction of nuclear deformation, favoring the opening of nuclear pores and the entry of YAP into the nucleus, with subsequent effects on key pathways like cell survival and differentiation. The knowledge of the precise extent of nuclear curvature required for YAP activation was still lacking until very recently, but Ghagre et al. used a FRET-based tension sensor to measure the nuclear membrane bending required for YAP activation in mesenchymal stem cells (MSCs) [60]. The authors found that there is a specific threshold of nuclear membrane curvature required for YAP activation. Below this critical point, which corresponds to a certain level of mechanical stress on the nucleus, YAP would exhibit a cytoplasmic localization, and its translocation into the nucleus would occur when the threshold is exceeded. Moreover, they showed that membrane curvature affects YAP localization by impacting its nuclear entry through both passive and active mechanisms. The passive import of YAP occurs via the simple diffusion through the nuclear pore complex due to its size, whereas the active import relies on YAP phosphorylation status and its subsequent interaction with the nuclear import machinery.

An important aspect of curvature sensing is its bidirectional interplay with curvature generation: cells not only detect curvature but also respond by modulating their cytoskeleton and membrane tension. This feedback loop is particularly evident in clathrin-mediated endocytosis (see “The impact of membrane curvature on endocytosis and exocytosis” section of this review), where curvature-sensing proteins stabilize membrane deformation, facilitating vesicle formation. However, at larger scales, the molecular mechanisms linking curvature sensing and generation remain less understood. One missing link is the role of mechanosensitive pathways in integrating curvature cues with intracellular signaling. For example, how do cells translate large-scale curvature changes into biochemical responses? Future research could explore how mechanosensitive ion channels, tension-dependent recruitment of curvature-sensing proteins, and force transmission through the cytoskeleton contribute to this process.

The interactions between nuclear curvature and other mechanical signals (e.g., substrate stiffness) are not yet fully understood. Interestingly enough, in a cancer model, Emon et al. showed that the degree of nuclear curvature was predominant over the tumor microenvironment stiffness in the regulation of YAP nuclear transport, which may have significant implications for understanding and potentially targeting mechanotransduction in cancer therapy [61].

### Cell differentiation

The ability of substrate curvature to favor cell differentiation is a key consideration in the field of tissue engineering and regeneration and has raised much interest (Fig. 3, lower part). This phenomenon has been studied across various cell types, including mesenchymal stem cells (MSCs) [43], neural [62], endothelial, epithelial [63], muscle, and bone cells (Table 2). RNA sequencing analysis of human MSCs revealed notable gene expression changes related to differentiation when cultured on sinusoidal (curved) surfaces compared to flat ones. These findings suggest that the curved topography of the surface influences stem cell behavior and differentiation pathways [2]. The underlying mechanisms by which bended surfaces influence cell differentiation likely involve a combination of changes in cell–matrix interactions, mechanotransduction signaling, intercellular contacts, and epigenetic regulation.

**Table 2:** Overview of curved surfaces used to enhance cell differentiation in vitro. Several examples of curvature-driven cell differentiation, selected from diverse physiological contexts, are presented, along with the corresponding cell lines, the specific phenotypes achieved, and the key differentiation markers detected

Cell lines	Curvature tested	Resulting phenotypes	Differentiation markers/activated pathways	References
Human mesenchymal stem cells (hMSCs) from the bone marrow stroma	Concave and convex surfaces with diameters ranging from 250 to 750 µm (larger than cell size) Principal curvature k = 1/175 µm^−1^	*Concave surfaces induce:* Long cell body extension Spider-like morphology Focal adhesion mostly in periphery Low number of focal adhesions Increase of migration speed *Convex surfaces induce:* Osteogenic differentiation Nuclear compression Snail-like configuration	Strong osteocalcin level on convex structure and higher lamin-A level	https://doi.org/10.1002/advs.201600347 Ref. [43]
Human mesenchymal stem cells (hMSCs)	Sinusoidal surfaces with 100 µm period and 10 µm amplitude (S10/100), as well as their homothetic counterparts 30 to 3 µm (S3/30) and 300 to 30 µm (S30/300)	Nucleus relaxation on concave curvature (→ quiescent state ?) on S10/100	Downregulation of Keratin7 (KRT7), paralemmin expressed in neurons (PALM) and transcription factors involved in differentiation processes (GLIS2, neuronal differentiation and kidney morphogenesis; CRIP2, differentiation of smooth muscle tissue; GPRC5A, embryonic development and cell differentiation; NGF, neuron growth), on S10/100 and S30/300	https://doi.org/10.1038/s41467-018-06494-6 Ref. [2]
Human adipose-derived mesenchymal stem cells (ADMSCs)	Honeycomb structure in 2D and 3D microenvironment Width: 20 µm Height: 60 µm	Honeycombs promote osteoblast generation	Honeycombs increase the expression of BSP and RUNX2, involved in osteogenesis	https://doi.org/10.1002/term.3108 Ref. [59]
Murine mesenchymal stem cells (MSCs)	Micropatterned lines Narrow pattern of 1.5 µm and wide patterns of 8 µm	Narrow pattern → high nuclear curvature and nuclear membrane tension Wider pattern → low nuclear curvature and nuclear membrane tension	MSCs confined in narrow patterns display increased nuclear YAP compared to those in wider patterns High nuclear curvature stimulates YAP nuclear translocation Low nuclear curvature favors adipogenic differentiation of MSCs with an increase of the ALP/Lpdx ratio	https://doi.org/10.1016/j.bpj.2024.04.008 Ref. [60]
Mouse N2a neuroblastoma cell line	Micropatterns with concave and convex shapes 1 cm × 1 cm × 1 cm (width × length × height)	Both concave and convex substrates trigger similar effects: Increase of neurite outgrowth Higher neurite complexity Neuronal polarity Higher nuclear displacement	Decrease of YAP translocation Increase of YAP, Tuj-1, and GFAP	https://doi.org/10.1007/s42242-023-00243-5 Ref. [62]
Human corneal stroma cells	Mounting curved surface in the center and flat surface at the periphery. 13 mm width × 3 mm height Cell were seeded on the flat surface	Self-organization, ECM deposition	High expression of the corneal stromal markers keratocan, lumican, decorin, ALDH3, CHTS6	https://doi.org/10.1002/adbi.201700135 Ref. [63]
Mouse C2C12 myoblasts	Curved hemicylinder-shaped surface	Curved surfaces promote myoblast alignment and differentiation	3–10 mm diameter hemicylinders decrease Pax7 level and increase expression of genes coding for myoblast determination and key factors for skeletal muscle	https://doi.org/10.1002/adbi.202000280 Ref. [64]
Human MG-63 osteosarcoma cells	2 µm grooves/6 µm ridges (2/6)	Alignment of the cells along grooves Osteogenic differentiation	Use of dexamethasone as biological stimulator of osteogenic differentiation Narrow grooves/ridges (2/6) increase expression of ALP, BMP-2, and Runx2	https://doi.org/10.1371/journal.pone.0161466 Ref. [56]
Human and mouse primed pluripotent stem cells (PSC)	Microstructure curvature ranging between 15 and 62 mm^−1^	Pluripotent stem cells (PSCs) reverted to the naive state on these substrates, indicated enhanced developmental potential, reflecting early embryonic development compared to their primed counterparts The upregulation of pluripotency markers like NANOG after the cells were detached from the substrate suggests that the cells had durably reverted to a naïve state	Increased E-cadherin/RAC1 signaling, along with activation of the nuclear effector YAP, was observed and resulted in histone modifications that promote the expression of naïve pluripotency markers	https://doi.org/10.1038/s41563-024-01971-4 Ref. [65]

To enhance the differentiation of myocytes, which are highly aligned cells displaying an elongated shape, researchers have cultured C2C12 myoblasts on parallel grooves, leading to increased expression of the determination factor MyoD1 as well as myocyte biomarkers like myosin heavy chain 3 (MYH3) and myogenin [64]. This suggests that morphological changes (e.g., elongation, alignment) induced by well-designed anisotropic curvature networks might mimic the native tissue microenvironment and stimulate both lineage determination and functional maturation. More precisely, curved landscapes can influence myoblast fusion, formation of multinucleated cells, which are characteristic features of muscle differentiation, as well as sarcomere assembly and contractile protein synthesis, leading to enhanced muscle cell differentiation.

Besides triggering differentiation-permissive morphological cues, curvature also induces some mechanical cues modulating differentiation. Several studies have underlined that cellular tension, adhesion, and mechanotransduction signaling pathways may be involved [45, 66, 67]. As already described for migration, some key cytoskeletal components are modified by curvature and can modulate differentiation. Indeed, substrate curvature can affect the distribution and activity of focal adhesion complexes, integrins, and other cell–matrix adhesion molecules, influencing the adhesion, spreading, and migration of osteoprogenitor cells [68]. Curvature can also affect cell-ECM interactions, matrix deposition, and mineralization, influencing the differentiation and activity of osteoblasts during bone formation. Cells cultured on curved substrates may exhibit altered cell–cell contacts, communication, and signaling, which can impact differentiation processes such as morphogenesis, tissue patterning, and organ development. Finally, curvature can regulate gene expression patterns associated with differentiation-related signaling pathways, lineage commitment, and cell fate determination. Building on this idea, Xu et al. recently developed a substrate mimicking the geometry of the blastocyst (i.e., concave surfaces with principal curvatures of opposite signs, resembling the curved environment of the early embryo), to induce naivety in pluripotent stem cells in vitro. This motif substrate, featuring specific curvature ranges (between 15 and 62 mm^−1^), enhances E-cadherin/RAC1 signaling and YAP activation, leading to increased expression of the pluripotency marker Nanog and improved developmental potential (Fig. 4). This discovery provides valuable insights into substrate design for promoting naivety and holds potential for regenerative medicine applications [65].

Altogether, these findings suggest that engineering the curvature of biomaterial scaffolds and substrates could be a valuable strategy to guide and control cell fate during tissue regeneration and engineering applications. It remains to be determined which precise pattern of curvatures may suit optimally for each cell type and specific purpose. Further elucidation of these curvature-mediated effects on cellular differentiation processes remains an active area of research in the field.

## Challenges and future perspectives

The study of curvature and its impact on biological systems is an emerging field that is gaining increasing attention, with a growing community of researchers contributing to its development and exploration. Among its many intriguing aspects is the potential to uncover how curvature directly and indirectly influences chromatin organization and the regulation of gene expression. High-throughput analyses such as RNA sequencing, assay for transposase-accessible chromatin using sequencing (ATAC-sequencing), and methylome profiling could offer valuable insights in this regard. Furthermore, the use of stem cell-derived organoids, as a groundbreaking model for 3D studies, will undoubtedly be of great interest for many aspects of curvature research. Additionally, recent advancements in micro- and nanofabrication techniques [69, 70], combined with progress in computational modeling [71, 72], have the potential to significantly enhance our understanding of curvature in biological systems, enabling the design of precise experimental systems and the development of predictive tools.

## Data Availability

No datasets were generated or analysed during the current study.

## Abbreviations

ATAC-sequencing: Assay for transposase-accessible chromatin using sequencing
AFM: Atomic force microscopy
BAR: Bin/amphiphysin/Rvs
BMP: Bone morphogenetic protein
CME: Clathrin-mediated endocytosis
ECM: Extracellular matrix
ERK: Extracellular signal-regulated kinase
FAK: Focal adhesion kinase
FRET: Förster resonance energy transfer
4D: Four dimension
GUVs: Giant unilamellar vesicles
GPCRs: G protein-coupled receptors
hMSCs: Human mesenchymal stem cells
LUVs: Large unilamellar vesicles
LINC: Linker of nucleoskeleton and cytoskeleton
MSCs: Mesenchymal stem cells
PDMS: Polydimethylsiloxane
QCM: Quartz crystal microbalance
SUVs: Small unilamellar vesicles
SPR: Surface plasmon resonance
3D: Three dimension
TIRF: Total internal reflection fluorescence microscopy
TAZ: Transcriptional co-activator with PDZ-binding motif
2D: Two dimension
YAP: Yes-associated protein
